# Application of Whisker-Toughened Aerogel to Recycling of Used Polyurethane Sheets

**DOI:** 10.3390/gels10120793

**Published:** 2024-12-04

**Authors:** Xiaohua Gu, Jiatong Chen, Shangwen Zhu, Qinglong Zhao, Yanxun Zhang, Qingyong Su

**Affiliations:** 1 School of Energy and Building Environment, Guilin University of Aerospace Technology, Guilin 541004, China; 2022043@guat.edu.cn (S.Z.); 2021008@guat.edu.cn (Y.Z.); sqy@guat.edu.cn (Q.S.); 2 Qiqihar School of Materials Science and Engineering, Qiqihar University, Qiqihar 161006, China; 3 Guangxi Key Laboratory of Green Building Materials and Construction Industrialization, Guilin University of Technology, Guilin 541004, China; 2120230846@glut.edu.cn; 4 College of Civil Engineering and Architecture, Northeast Petroleum University, Daqing 163318, China; 238003050924@stu.nepu.edu.cn

**Keywords:** waste polyurethane sheets, mullite whiskers, SiO_2_ aerogel, whisker toughening, composite materials

## Abstract

In this study, a new environmentally friendly and efficient method for recycling and reusing waste polyurethane sheets is proposed. SiO_2_ aerogel was prepared using the sol–gel method, and mullite whiskers were introduced to enhance its toughness. The whisker-toughened aerogel was used in the degradation of waste polyurethane to produce modified recycled polyol, which was subsequently used to prepare recycled polyurethane foam insulation material. Following a series of tests, including Fourier-transform infrared spectroscopy, apparent density, viscosity, heat loss, and thermal conductivity, the results showed that when the aerogel with wt% = 0.9% mullite whiskers and 0.06 g of whisker-toughened aerogel were added, the viscosity was close to that of polyether polyol 4110. The optimal compressive strength of the resulting composite blister structure reached 817.93 MPa, with a thermal conductivity of 0.0228 W·(m·K)^−1^, demonstrating good thermal stability. These results indicate that the whisker-toughened aerogel effectively reduces the viscosity of the degraded materials and significantly improves the mechanical properties and thermal stability of the regenerated polyurethane thermal insulation materials. This research provides new ideas and new methods for waste polyurethane recycling and offers a new perspective for the research and development of thermal insulation materials.

## 1. Introduction

Polyurethane foam sheets are thermosetting polymers that are renowned for their exceptional thermal insulation properties, with thermal conductivities that typically range between 0.02 and 0.03 W/(m·K) [1]. This low thermal conductivity ensures equivalent thermal insulation effects and also results in less material consumption compared to that of other insulation materials, thereby effectively increasing the actual building area [2]. Consequently, polyurethane foam has become a staple in the construction industry for insulation purposes. However, the recycling of waste polyurethane foam panels presents significant challenges [3,4]. The traditional physical recycling methods, which involve mechanical processes such as crushing and screening, are simple and cost-effective, but suffer from low material recovery and utilization rates [5,6,7]. Energy recovery methods such as incineration convert waste polyurethane into heat or other forms of energy, but these approaches are energy-intensive and can lead to environmental pollution [8,9]. Chemical recycling, a widely adopted alternative, modifies the molecular structure of polyurethane through chemical reactions, such as alcoholysis, alkaline digestion, and hydrolysis, allowing the material to be reprocessed into new products. This method better maintains the material’s performance, although it requires precise control over the reaction conditions to ensure degradation efficiency and product quality. Despite these challenges, chemical recycling boasts superior environmental adaptability and enhanced resource recovery efficiency; thus, it is in alignment with the sustainable development goals [10,11,12].

This study focuses on SiO_2_ aerogel, which is a novel building material with unique thermal insulation properties that give it great potential in the field of building energy efficiency [13]. However, the high porosity of SiO_2_ aerogel leads to its insufficient mechanical properties; consequently, its application scope is limited [14,15,16,17]. To solve this problem, we adopted the whisker-toughening technique and introduced mullite whiskers [18,19,20,21,22] to enhance the mechanical properties of SiO_2_ aerogel and to improve its thermal and chemical stability [23,24,25,26]. On this basis, we explored the application of whisker-toughened SiO_2_ aerogel in the recycling of used polyurethane sheets [27,28]. By applying whisker-toughened aerogel to the degradation process of waste polyurethane, we successfully prepared modified recycled polyurethane materials. This approach not only improved the mechanical properties of the recycled polyurethane materials, but also maintained their excellent thermal insulation properties.

The innovative approach of this study reduces polyurethane foam waste and also produces recycled polyurethane foam composites with an excellent performance. Our work increases the value of the product; it is also important for reducing carbon emissions and promoting energy conservation. Through this study, we provide a new strategy for the efficient recycling and reuse of waste polyurethane panels and new ideas for the development of energy-saving materials for buildings.

## 2. Results and Discussion

### 2.1. Analysis of Whisker-Toughened SiO_2_ Aerogel Materials

#### Scanning Electron Microscopy Analysis of Whisker-Toughened SiO_2_ Aerogel

**Figure 1:**
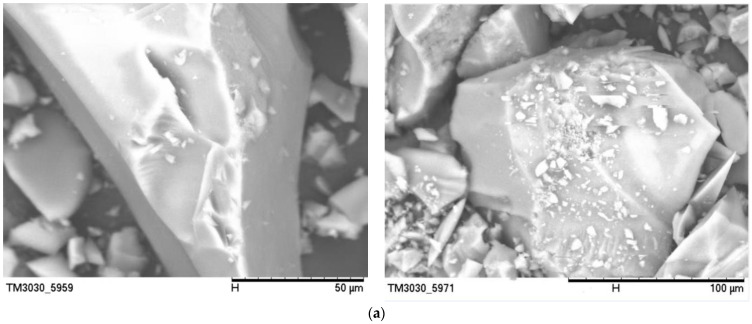

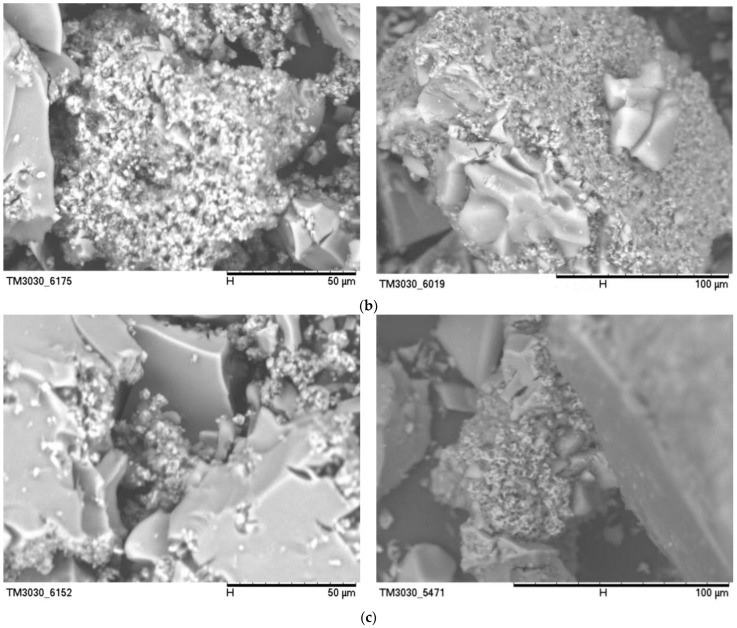
Scanning electron microscopy of SiO2 aerogel with different whisker contents: (**a**) whisker wt% = 0.6%; (**b**) whisker wt% = 0.9%; (**c**) whisker wt% = 1.2%.

The results of the whisker-toughened SiO_2_ aerogel scanning electron microscopy tests are shown in Figure 1.

As can be seen in the figure, the microstructure of silica aerogel shows a three-dimensional network skeleton. Silica particles can reach the nanometer level, and these silica particles pile up on top of each other to form a skeleton structure. When the whisker content rises, the network skeleton of the aerogel experiences a certain type of collapse due to agglomeration. The three-dimensional network structure of the silica aerogel product has a greater possibility of fracture; consequently, the distribution of silica particles ceases to be uniform, and the performance of the silica aerogel is affected. From the following microstructure observation and the strength test results of the composites, it is also shown that 2# aerogel with wt% = 0.9% doping has a better vesicle structure and a compression strength up to 817.93 MPa. Therefore, wt% = 0.9% is a reasonable whisker content.

### 2.2. Analysis of Whisker-Toughened SiO_2_ Aerogel-Regenerated Polyol-Based Polyurethane Nanocomposites

#### 2.2.1. Fourier-Transform Infrared Spectral Analysis of Whisker-Toughened SiO_2_ Aerogel-Regenerated Polyol-Based Polyurethane Nanocomposites

**Figure 2:**
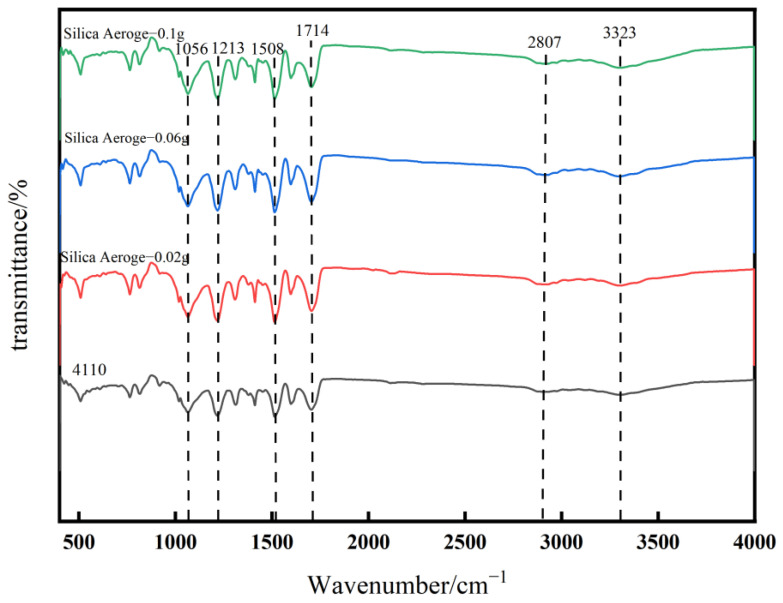
Infrared spectra of recycled polyurethane foam with different silica aerogel additions.

The infrared spectra of the regenerated polyurethane nanocomposites of the samples with pure aerogel added, and then foamed with polyether 4110 and those with different silica aerogel additions are shown in Figure 2.

In Figure 2, the infrared spectra of the regenerated polyurethane foam with and without different silica–mullite whisker composite aerogel additions are approximately the same, with some differences only at some vibrational peaks. As can be seen in the figure, the telescopic vibrational peaks appear as hydroxyl and N–H near 3323 cm^−1^. There is a stretching vibration peak of C–H in methyl or methylene at 2807 cm^−1^ and a characteristic peak of –NCO at 2255 cm^−1^, which indicates that the reaction is more complete without a large amount of unreacted material remaining due to the extremely small peak. The peak appearing at 1714 cm^−1^ is the vibrational peak of the C=O bond in the carbamate group; the peak appearing at 1508 cm^−1^ is the stretching vibrational peak of –NH. There is a more pronounced peak at 1213 cm^−1^ for the stretching vibrational peak of C–N, and the stretching vibrational peak of the ether bond is found at 1056 cm^−1^. The bending vibrational peaks of C–H and the O–Si–O bending vibration peaks are between 758 and 823 cm^−1^, and it can be seen in the figure that the peaks of the added aerogel materials are more obvious. In summary, it can be seen that the infrared spectra of the regenerated polyurethane foam prepared using recycled polyol and the addition of silica–mullite whisker composite aerogel are the same as those of polyurethane foam prepared using polyether 4110; this indicates that the specimen prepared using regenerated polyol is polyurethane foam and that the addition of silica–mullite whisker composite aerogel did not destroy the main structure [29].

#### 2.2.2. Viscosity Test Analysis of Waste Polyurethane Degradation Products

The results of viscosity testing and the analysis of the waste polyurethane degradation products are shown in Table 1.

According to the weights of the products shown in Table 1, there may be undegraded foam in the waste foam alcohol when 1# aerogel is used. From the viscosity point of view, viscosity resulting from the use of 2# aerogel is relatively the lowest. In this case, adding 0.06 g results in the closest viscosity to that of polyether 4110 measured in the laboratory, which is 3583.6 m·Pa·s; thus, it is judged to have an optimal viscosity value of 3864.52 mPa.s. There are more degradation products when 3# aerogel is used, but they have high viscosity. Considering the weight and viscosity of the products, 2# aerogel has the best degradation performance with the lowest viscosity when adding 0.06 g.

#### 2.2.3. Scanning Electron Microscope Test and Analysis of Whisker-Toughened SiO_2_ Aerogel-Regenerated Polyol-Based Polyurethane Nanocomposites

**Figure 3:**
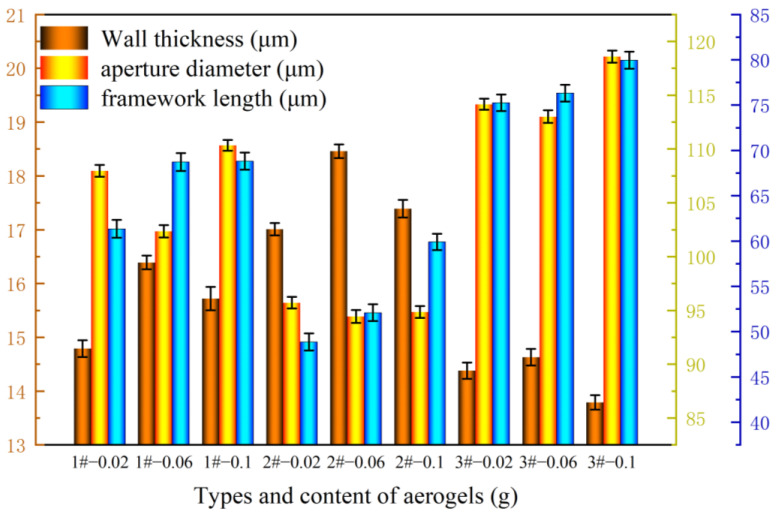
Wall thickness, pore size, and skeleton length of recycled polyurethane foams prepared using silica aerogel with different compositions and additions.

**Figure 4:**
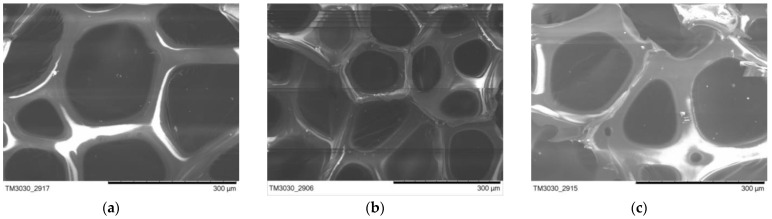
Micrographs of recycled polyurethane foams prepared with different additive levels: (**a**) 0.02 g; (**b**) 0.06 g; (**c**) 0.1 g.

The results of the whisker-toughened SiO_2_ aerogel-regenerated polyol-based polyurethane nanocomposites analyzed using scanning electron microscopy tests are shown in Table 2 and Figure 3 and Figure 4.

From the test results, it can be seen that the vesicles of the regenerated polyurethane foam nanocomposites prepared using different silica aerogels are relatively complete without large-scale breakage phenomena. When the added mass of silica aerogel is 0.06 g, the vesicles of the regenerated polyurethane foam are more complete and close to a pentagonal shape. When the added amount of silica aerogel is increased, the vesicles are occasionally ruptured due to the phenomenon of the agglomeration of the aerogel, and the pore walls become thinner. The closure of the foam vesicles at this time is poor, and the strength is reduced. With the addition of too much silica aerogel, agglomeration affects the formation of bubble holes. The degree of cross-linking of the foam and the reduction in strength of silica aerogel also affect the formation of the bubble hole film; consequently, the bubble holes rupture, and strength is reduced [30]. The agglomeration of silica aerogel also affects the formation of the vesicle membranes, causing the vesicles to rupture and affecting the performance of the regenerated polyurethane foam. Therefore, when the added amount of silica aerogel is 0.06 g, the nanocomposite of the regenerated polyurethane foam achieves the best performance.

#### 2.2.4. Analysis of Apparent Density Testing of Whisker-Toughened SiO_2_ Aerogel-Regenerated Polyol-Based Polyurethane Nanocomposites

**Figure 5:**
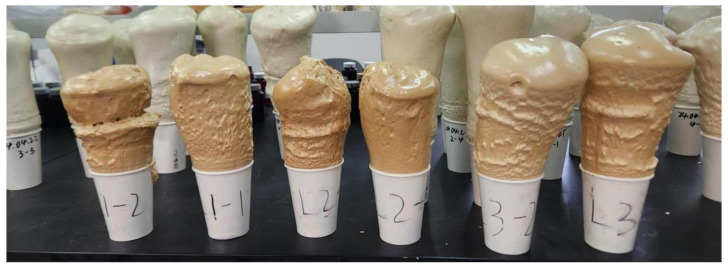
Samples of recycled polyurethane foam prepared with different silica aerogel additions (from left to right, with two each of 0.02 g, 0.06 g, and 0.1 g).

**Figure 6:**
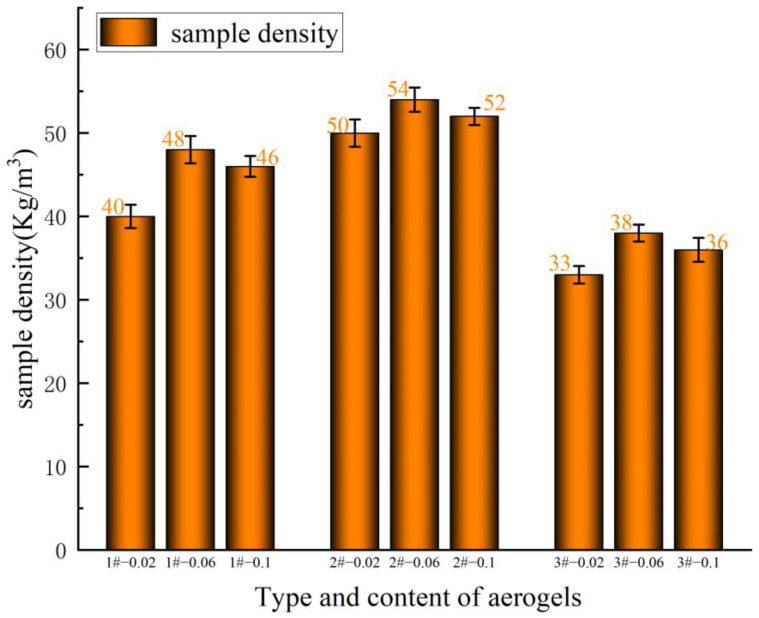
Density of recycled polyurethane foam samples prepared with different silica aerogel additions.

Figure 5 shows the experimental whisker-toughened aerogel-regenerated polyol-based polyurethane nanocomposites, and Figure 6 shows the results of the apparent density test of the regenerated polyurethane, from which it can be seen that the regenerated polyurethane foam specimen with the addition of silica aerogel has a minimum density of 33 kg/m^3^. Due to the three-dimensional mesh structure of the silica aerogel becoming a part of the polyurethane skeleton, there is an increase in the pore size of the bubbles, resulting in a small increase in the density of the regenerated polyurethane foam. The density of the recycled polyurethane foam increases slightly. As the amount of silica aerogel increases, some silica aerogel particles may form an agglomeration; consequently, the fluctuation in the regenerated polyurethane nanocomposites is larger. When the amount of silica aerogel added to the regenerated polyurethane nanocomposites is 0.06 g, the density fluctuations are the smallest, and density reaches a maximum of 54 kg/m^3^.

#### 2.2.5. Test and Analysis of Compressive Strength of Whisker-Toughened SiO_2_ Aerogel-Regenerated Polyol-Based Polyurethane Nanocomposites

**Figure 7:**
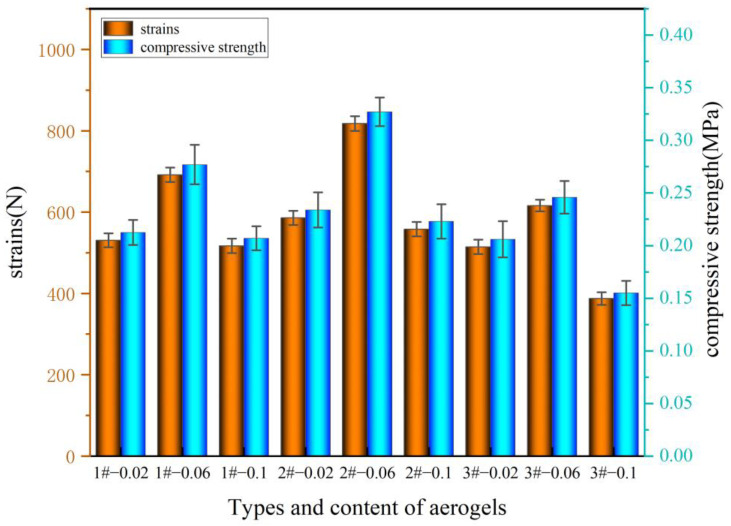
Compressive strengths of different regenerated polyurethanes prepared from silica aerogels with different compositions and contents.

The compressive strengths of the samples are shown in Table 3 and Figure 7.

The strength of the pure polyurethane specimen is 0.125 MPa, and the addition of aerogel increases the strength. The maximum strength appears with the addition of 0.06 g of aerogel, at a value of 0.3271. This is because of the reasonable content range of aerogel and the comparably high strength of polyether polyol polymerization. The addition of aerogel content for aerogel strength has a greater impact on the aerogel, and there is a low impact of about 0.2 MPa on the aerogel’s strength [31].

#### 2.2.6. Test and Analysis of Thermal Conductivity of Whisker-Toughened SiO_2_ Aerogel-Regenerated Polyol-Based Polyurethane Nanocomposites

Polyurethane foam is commonly used as an insulating material in daily life; thus, thermal conductivity is an important index for polyurethane foam. Due to the low thermal conductivity of the aerogel, as well as its better thermal insulation and heat preservation performance, its addition to the preparation of recycled polyurethane can bring out the corresponding characteristics; consequently, the prepared recycled polyurethane foam has better performance. The thermal conductivity of the recycled polyurethane prepared with different contents of silica aerogel is shown in Table 4.

As can be seen in Table 4, the thermal conductivity of each of the recycled polyurethane foam nanocomposites complies with the national standard of less than 0.036 W/(m·K). Compared to the recycled polyurethane foam without silica aerogel, the addition of silica aerogel results in the lower thermal conductivity of the recycled polyurethane foam nanocomposites. With the increase in silica aerogel addition, the overall trend of thermal conductivity decreases, indicating that silica aerogel has an obvious optimization effect on the thermal conductivity of the regenerated polyurethane foam nanocomposites; the thermal insulation performance is improved, and it is therefore possible to create better thermal insulation materials. When the amount of added silica aerogel reaches 0.06 g, thermal conductivity does not change much, and the thermal insulation effect is better. When the amount of silica aerogel is further increased to 0.1 g, the thermal conductivity of the regenerated polyurethane foam increases, and the strength also begins to decline; this proves that the aerogel produces an agglomeration phenomenon, resulting in a decline in the thermal insulation performance. Therefore, a 0.06 g addition of aerogel is the optimal amount of additive to produce better whisker-toughened aerogel-regenerated polyol-based polyurethane nanocomposites.

#### 2.2.7. Analysis of Whisker-Toughened SiO_2_ Aerogel-Regenerated Polyol-Based Polyurethane Nanocomposites for Heat Loss Testing

**Figure 8:**
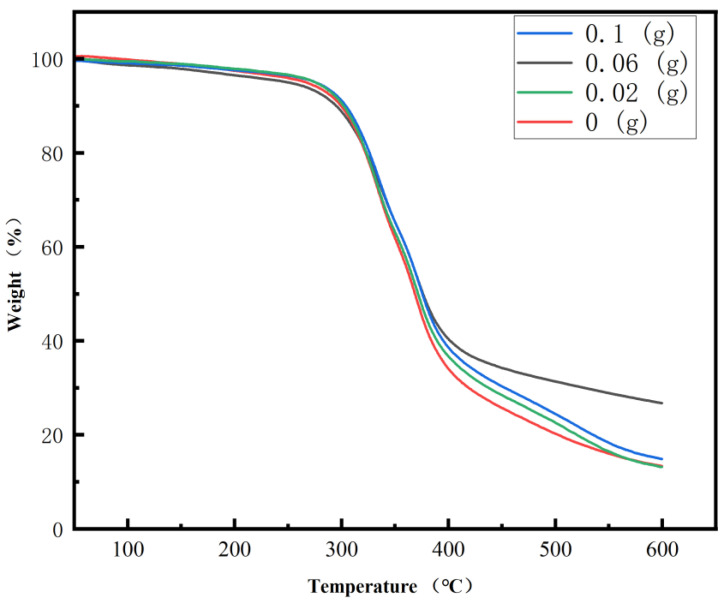
Heat loss spectra of recycled polyurethane foams with different silica aerogel additions.

The heat loss spectra of the regenerated polyurethane foam nanocomposites with different silica aerogel additions are shown in Figure 8. From the spectra, it can be seen that the heat weight loss process of the regenerated polyurethane foam can be divided into three stages. The first stage is 0–300 °C, which is mainly the water loss stage of the regenerated polyurethane foam; the weight of the regenerated polyurethane foam has a weak reduction, but there is no obvious difference. The second stage has a temperature range of 300–400 °C. With the addition of silica aerogel, the decomposition temperature of the regenerated polyurethane foam reaches 325 °C. It can be seen that silica aerogel will improve the thermal stability of the regenerated polyurethane foam. The third stage is 400–600 °C; in this stage, it can be clearly seen that in the regenerated polyurethane foam prepared with the addition of 0.06 g of silica aerogel, the residual rate is significantly higher than that of the other components. Furthermore, the rate of heat weight loss also has a significant downward trend, proving that the component has the best thermal stability [32].

### 2.3. Discussion and Analysis

#### 2.3.1. Mechanism of Whisker-Toughened Silica Aerogel-Reinforced Polyurethane Foam

**Figure 9:**
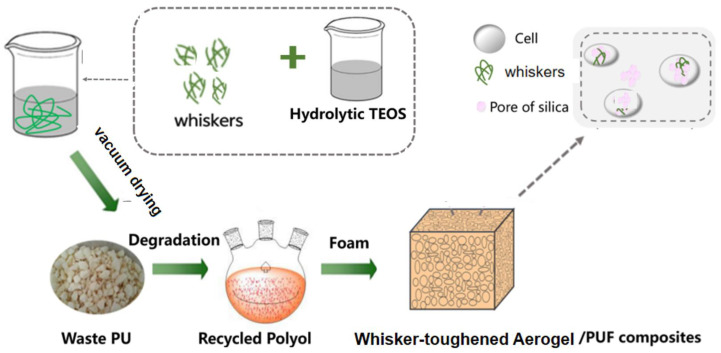
Mechanism of whisker-toughened silica aerogel-reinforced polyurethane foam.

The whisker-toughened silica aerogel-reinforced polyurethane foam mechanism is shown in Figure 9. The mechanism of whisker-toughened silica aerogel-reinforced polyurethane foam mainly involves the following aspects. Firstly, the whiskers of whisker-toughened aerogel play the role of bridging and transferring stress in aerogel materials; when the material is subjected to external force, the whiskers can effectively disperse and transfer the stress to improve the toughness of the composite material. The whiskers can also inhibit the expansion of cracks, as their high strength and high modulus characteristics prevent the propagation of cracks in the aerogel material. Secondly, whisker-toughened silica aerogel has a synergistic enhancement effect due to the fact that the whisker-toughened silica aerogel surface contains silica hydroxyl. When aerogel is added to the diol, a part of the silica aerogel silica hydroxyl will replace the diol’s hydroxyl. The alcohol solvent, the diol solvent, and whisker-toughened silica aerogel hydroxyl have a synergistic effect. In the catalyst role, the solvent degradation process usually occurs at the interface between the polyurethane material and the solvent, and gradually extends internally as the solvent penetrates, leading to the degradation of the polyurethane through the dissolution of specific urethane groups in the polyurethane molecular chain of the breaking group or the destruction of intermolecular chain interaction forces. In the presence of an alcohol and a catalyst, the urethane’s urethane groups break. After fracture, the short alcohol chain of diol and aerogel will replace the original long chain; as the reaction proceeds, the polyurethane molecular chain is gradually broken to form an oligomer, releasing the long-chain polyol and aromatic compounds to form the degradation product. The regenerated polyurethane foam whisker-toughened aerogel obtained by the degradation product can be used as the foam skeleton to play a supportive role; in addition, the nanometer-sized pore structure of the aerogel and the whisker’s high aspect ratio can improve the performance of the composite material. Furthermore, the nanoscale pore structure of the aerogel and the high aspect ratio of the whiskers can improve the performance of the composites. This can also be seen in thermal conductivity analysis and heat loss analysis in this study; the thermal conductivity of the whisker-toughened aerogel group decreased up to 0.0069 W/(m·K) compared with that of the pure sample group, and thermal stability was also improved [33,34].

#### 2.3.2. Heat Insulation Mechanism of Whisker-Toughened Silica Aerogel-Regenerated Polyol-Based Polyurethane Nanocomposites

**Figure 10:**
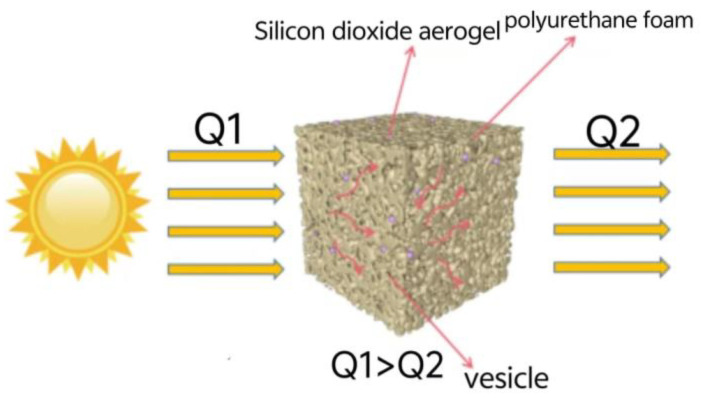
Heat insulation mechanism of whisker-toughened silica aerogel-regenerated polyol-based polyurethane nanocomposites.

As shown in Figure 10, the heat insulation mechanism of the silica aerogel-regenerated polyol-based polyurethane nanocomposites, the silica aerogel, and the vesicles blocked the gas and heat transfer pathway, causing the heat transmission channel to become longer. Therefore, silica aerogel-regenerated polyol-based polyurethane nanocomposites have a better thermal insulation effect, minimizing the loss of heat. In addition, the aerogel separates the bubble holes into smaller spaces, and the aerogel itself becomes part of the porous material, which further improves the material porosity and causes thermal conductivity to decrease. The silica aerogel-regenerated polyol-based polyurethane nanocomposites have good thermal insulation performance [35,36].

## 3. Conclusions

In this study, the introduction of mullite whiskers into the process of silica aerogel preparation successfully achieved the toughening of the aerogel. The obtained whisker-toughened silica aerogel was used in the recycling of waste polyurethane, and the obtained regenerated polyurethane foam was tested and analyzed. It was found that the whisker-toughened aerogel-regenerated polyurethane thermal insulation material had an excellent thermal insulation performance, and it could significantly reduce the energy consumption of the building. In addition, it had better environmental adaptability. The specific performance is as follows:

1. With a wt% = 0.9% whisker content, the distribution of silica aerogel particles is uniform, and the three-dimensional network skeleton structure is optimal.
2. Used polyurethane was prepared for recycling using a wt% = 0.9% whisker content with 0.06 g of 2^#^ aerogel. The waste polyurethane degradation had the lowest viscosity of 3864.52 m·Pa·s; this was produced by the regeneration of polyurethane foam. The foam hole wall thickness was 18.46, the pore diameter was 94.45, and the length of the skeleton was 52.09. The bubble skeleton was thick and had a sturdy structure that was close to ortho-pentagonal. The physical properties were good, with a density of 54 kg/m^3^ and a compression strength of up to 0.3271 Mpa.
3. The thermal properties resulting from the use of wt% = 0.9% whisker content with 0.06 g of 2^#^ aerogel were examined in recycled polyurethane. With a thermal conductivity of 0.0228 W/(m·K), the insulation effect was good. At the thermal decomposition temperature of 275 °C, the residual rate of thermal stability, heat preservation, and the thermal insulation performance were excellent.

## 4. Materials and Methods

### 4.1. Whisker-Toughened Aerogel Preparation

The raw materials and reagents used in this part of the experiment are shown in Table 5.

**Figure 11:**
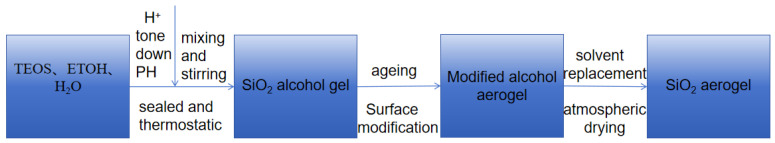
Mechanism of silica aerogel preparation using sol–gel method.

Whisker-toughened aerogel was prepared using the sol–gel method. The reaction principle is shown in Figure 11. Ethyl orthosilicate, anhydrous ethanol, and deionized water were added into a beaker. Then, dilute hydrochloric acid was added dropwise, while the solution was stirred to adjust the pH so that it was within the range of 3–5. The stirring of the solution continued until the temperature no longer changed, which indicated that the reaction was complete. The solution was divided into three equal parts, and mullite whiskers with wt% = 0.6%, 0.9%, and 1.2% were added, respectively, to each part. Stirring was carried out until the whiskers were uniformly dispersed. Next, dilute ammonia was added dropwise to adjust the pH to neutral. The stirred solution was placed in a 40 °C constant-temperature water bath to form a wet gel. The wet gel was then immersed in a mixture of anhydrous ethanol and ethyl n-silicate for aging. After aging for 24 h, the gel was surface-modified with a mixture of KH550 and n-hexane, soaked in n-hexane for 24 h, and then filtered; this process was repeated three times. Finally, the gel was placed in a vacuum drying oven and dried at 60 °C for 6 h. The white solid obtained was the whisker-toughened aerogel, which was then finely ground to about 1 mm using a ball mill to ensure better dispersion.

### 4.2. Preparation of Whisker-Toughened Aerogel-Regenerated Polyol-Based Polyurethane Nanomaterials

The raw materials and reagents used in this part of the experiment are shown in Table 6.

A waste polyurethane sheet was used as the raw material; di-ethylene glycol and ethylene glycol were used as alcoholysis agents. Following cleaning and the drying treatment and crushing, the waste polyurethane sheet weighed 100 g. Then, 100 g of alcoholysis agent, of which ethylene glycol/di-ethylene glycol = 43:57, and 0.8 g of the catalyst NaOH and a certain quality of the aerogel (as an additive, as shown in Table 7) were added to the three reaction kettles. Whisker wt% = 0.6% was added to aerogel group 1#; whisker wt% = 0.9% was added to aerogel group 2#; whisker wt% = 1.2% was added to aerogel group 3#. The device was placed into a thermoregulation electric heating jacket, which was set to 120 °C; it was then heated and stirred for about 20 min. Then, 100 g of waste polyurethane powder was added to the three reaction kettles, and the temperature was increased to 190 °C; it was stirred for about 2 h, and the regenerated polyols were poured out when the temperature had reduced to room temperature.

A total of 10 g of regenerated polyols, 20 g of commercial polyether polyol 4110, 2.5 g of dimethicone, 13 g of foaming agent, 0.3 g of the catalyst triethanolamine (TEA), and 0.15 g of dibutyltin dilaurate (DBTDL) were added to a disposable plastic cup and mixed using a cantilever mixer at a mixing rate of 1000 rpm until well blended. Then, 45.5 g of the black material was added and mixed thoroughly. A cantilever mixer was used to mix at 1500 rpm until foam was formed. The resulting whisker-toughened, aerogel-regenerated, polyol-based polyurethane nanocomposites were placed in a cool place for 24 h, and then tested.

### 4.3. Test Methods

#### 4.3.1. Fourier-Transform Infrared Spectroscopy Analytical Tests

The recycled polyurethane foam prepared in Section 2.2 was analyzed using a Fourier-transform infrared spectrometer (PE, Waltham, MA, USA).

#### 4.3.2. Scanning Electron Microscope Tests

The whisker-toughened SiO_2_ aerogel and polyurethane foam slices were observed using a Zeiss Sigma 300 model scanning electron microscope from Zeiss, Oberkochen, Germany. (Zeiss Sigma 300, Carl Zeiss AG, Oberkochen, Germany). The samples were fixed with conductive adhesive on a carrier stage before the test; then, they were subjected to the surface spraying of gold, and the morphological condition of the samples was observed after spraying treatment.

#### 4.3.3. Viscosity Test

The degradation product was placed at room temperature in order to perform a viscosity test. The viscosity range was pre-estimated before measurement, a suitable rotor was selected, and the level of the instrument was adjusted. The level bubble was checked to ensure that it was centered. The bottle containing degradation product was placed directly under the probe of the viscosity tester, and the right handwheel for lifting and lowering was slowly adjusted. The rotor was immersed into the sample up to the mark of the groove on the rotor bar. The viscosity test was carried out, and the rotor rotational speed and the viscosity were recorded.

#### 4.3.4. Apparent Density Test

The whisker-toughened SiO_2_ aerogel-regenerated polyurethane nanocomposite specimens with different compositions and different silica aerogel additions were allowed to stand in a cool place for 72 h. Then, the regenerated polyurethane specimens were cut into 50 mm × 50 mm × 50 mm pieces to test the apparent density of the specimens. After they were weighed using an electronic balance; the apparent densities of the specimen pieces were calculated according to the formula ρ = m/V. This experiment was repeated 3 times for each group of formulations, and the average value was taken.

#### 4.3.5. Compression Strength Test

The instrument used was the HKW 50 kN universal material testing machine. The test sample was prepared as a square of 50 mm × 50 mm × 50 mm; its compressive strength was tested when deformation reached 10% under the condition of 20 mm/min displacement of the beam of the instrument. The tests of three identical samples were repeated, and the average value was taken.

#### 4.3.6. Thermal Conductivity Test

The size of the sample was 200 mm × 200 mm × 20 mm; it was tested using the FEHC-S thermal conductivity tester from China Changzhou Hua’ao Instrument Manufacturing Co., Changzhou, China.

#### 4.3.7. Thermal Weight Loss Test

A thermogravimetric analyzer was used to raise the temperature of the regenerated polyurethane test samples from a room temperature of 25 °C to 500 °C in nitrogen atmosphere at an elevated rate of 30 °C. For the analysis of heat loss, a gas flow rate of 50 mL/min at the level of air carrier gas was used, with alumina as a reference.

## Figures and Tables

**Table 1:** Viscosity of waste polyurethane degradation products.

Aerogel Types	Aerogel Content (g)	Rotors	Product Weigh (g)	Stickiness (m·Pa·s)	Rotational Speed (rpm)	Temp (°C)
1#	0.02	2#	163.9	6789.69	0.6	25.6
1#	0.06	2#	156.2	8562.36	6	25.6
1#	0.1	2#	172.5	8453.39	6	25.6
2#	0.02	3#	170.26	4356.56	12	25.6
2#	0.06	2#	183.47	3864.52	6	25.6
2#	0.1	2#	174.04	3968.34	0.6	25.6
3#	0.02	2#	164.8	6568.38	6	25.6
3#	0.06	2#	161.44	8846.95	6	25.6
3#	0.1	2#	161.43	8954.51	12	25.6

**Table 2:** Wall thickness, pore size, and skeleton length of recycled polyurethane foam.

Aerogel Types	Aerogel Content (g)	Wall Thickness (μm)	Diameter of Hole (μm)	Skeleton Length (μm)
1#	0.02	14.79	107.98	61.35
1#	0.06	16.39	102.35	68.72
1#	0.1	15.72	110.34	68.81
2#	0.02	17.01	95.72	48.86
2#	0.06	18.46	94.45	52.09
2#	0.1	17.39	94.85	59.91
3#	0.02	14.38	114.16	75.27
3#	0.06	14.63	113.01	76.32
3#	0.1	13.79	118.59	79.96

**Table 3:** Compression strength test results of recycled polyurethane samples.

Aerogel Types	Aerogel Content (g)	Maximum Pressure (Pa)	Compressive Strength (MPa)
1#	0.02	530.69	0.2125
1#	0.06	691.89	0.277
1#	0.1	517.1	0.207
2#	0.02	585.86	0.234
2#	0.06	817.93	0.3271
2#	0.1	557.95	0.223
3#	0.02	514.56	0.206
3#	0.06	616.16	0.246
3#	0.1	387.44	0.155

**Table 4:** Thermal conductivity of different recycled polyurethanes prepared with different contents of silica aerogel.

Silicon Dioxide Aerogel Addition (g)	Thermal Conductivity W/(m·K)
0	0.0297
0.02	0.0244
0.06	0.0228
0.1	0.0229

**Table 5:** Main reagents of the experiment.

Name of Reagents	Specifications and Grades	Manufacturer
Ethyl orthosilicate	Analytically pure	Shanghai McLean Biochemical Technology Co., Shanghai China
Anhydrous ethanol		Jinzhou Gucheng Chemical Reagent Co., Jinzhou, China
HCl		Maoming Xiongda Chemical Co., Maoming, China
Ammonia		Guangzhou Hoying Chemical Technology Co., Guangzhou, China
Silane coupling agent (KH550)		Jinan Haowen Industrial Co., Jinan, China
n-hexane		Shenzhen Baishunxing New Material Co., Shenzhen, China
Mullite whiskers	-	Self-restraint

**Table 6:** Whisker-toughened aerogel-regenerated polyol-based polyurethane preparation materials.

Name of Reagents	Specifications and Grades	Manufacturer
Polyether 4110	Industrial grade	Wanke New Material Co., Dongying, China
Triethanolamine (TEA)	Analytically pure	Nanjing Renheng Chemical Co., Nanjing, China
Dibutyltin dilaurate	Industrial grade	Shinden Chemical Materials (Shanghai) Co., Shanghai, China
Dimethyl silicone oil CAS287-92-3	Analytically pure	Jinan Silicon Harbor Chemical Co., Jinan, China
Black material (IPDI)	Industrial grade	Liang New Material Technology Co., Zibo, China
Cyclopentane	Analytically pure	Jinhe Chemical Co., Yantai, China
Whisker-toughened SiO_2_ aerogel	-	Self-restraint
Regenerated Polyol	-	Self-restraint

**Table 7:** Whisker-toughened aerogel additions.

Serial Number	1# (g)	2# (g)	3# (g)
1	0.02	0	0
2	0.06	0	0
3	0.1	0	0
4	0	0.02	0
5	0	0.06	0
6	0	0.1	0
7	0	0	0.02
8	0	0	0.06
9	0	0	0.1

## Data Availability

The original contributions presented in the study are included in the article, further inquiries can be directed to the corresponding author.
